# Fibular strut allograft influences reduction and outcomes after locking plate fixation of comminuted proximal humeral fractures in elderly patients: a retrospective study

**DOI:** 10.1186/s12891-019-2907-3

**Published:** 2019-11-03

**Authors:** Xueliang Cui, Hui Chen, Binbin Ma, Wenbin Fan, He Li

**Affiliations:** 1 Department of Orthopaedics, Zhongda Hospital, Southeast University, 87 Ding Jia Qiao, Nanjing, Jiangsu 210009 People’s Republic of China; 2 Trauma Center, Zhongda Hospital, Southeast University, 87 Ding Jia Qiao, Nanjing, Jiangsu 210009 People’s Republic of China; 3 Orthopaedic Trauma Institute (OTI), Southeast University, Nanjing, Jiangsu 210009 People’s Republic of China

**Keywords:** Proximal humeral fracture, Fibular allograft, Locking plate, Elderly patients

## Abstract

**Background:**

Proximal humeral fractures (PHFs) are the third most commonly occurring fractures in elderly patients. Most of these fractures can be treated with conservative methods, but the optimal surgical treatment strategy for unstable fractures in elderly patients remains controversial. This study aimed to compare the radiological and clinical outcomes between locking compression plate (LCP) fixation and LCP fixation with fibular allograft implantation for the treatment of comminuted PHFs.

**Methods:**

We retrospectively reviewed 60 patients (mean age, 72.75 years) with closed 3- or 4-part fractures, and a minimum of 2 years of follow-up. Fracture reduction was quantitatively determined by humeral head height (HHH) and neck-shaft angle (NSA). The clinical outcome was evaluated by Constant-Murley score (CMS) and American Shoulder and Elbow Surgeons (ASES) score.

**Result:**

The average radiological changes were higher in the LCP group than in the locking plate with fibular allograft group (HHH of 4.16 mm vs 1.18 mm [*p* < 0.001] and NSA of 9.94° versus 3.12° [p < 0.001]) . The final average outcome scores were lower in the LCP group than in the FA group (CMS of 73.00 vs 78.96 [*p* = 0.024] and ASES score of 72.80 vs 78.64 [*p* = 0.022]). The FA group showed better forward elevation (*p* = 0.010) and abduction (*p* = 0.002); however, no significant differences were observed for shoulder external rotation or internal rotation. The number of complications was higher in the LCP group (28.57%) than in the FA group (1.2%) (*p* < 0.001).

**Conclusion:**

For comminuted PHFs in elderly patients, LCP fixation combined with a fibular allograft is reasonable option to ensure satisfactory radiological and clinical outcomes.

**Trial registration:**

ZDYJLY(2018)New-9. Name of registry: IEC for clinical Research of Zhongda Hospital, Affiliated to Southeast University. Date of registration: 2018-05-17.

## Background

Proximal humeral fractures (PHFs) are common in elderly patients with osteoporosis [1]. The prevalence of PHFs is rapidly increasing because of an increasing elderly population worldwide [2]. Most of these fractures are non-displaced and can be treated with conservative methods. However, the optimal surgical treatment of unstable, displaced two-, three- and four-part fractures in geriatric patients remains controversial [3].

Locking plate fixation is currently the most widely used method for the management of unstable PHFs [4]. However, achieving stable fixation and maintaining intraoperative reduction in elderly patients with low bone mineral density is still difficult [5]. Complications including screw perforation and varus malalignment are often reported [6, 7]. Establishment of the medial column support is well known to achieve successful clinical outcome and reduce the complication rate [8, 9]. Various techniques have been advocated to reduce and enhance the medical column, such as cement augmentation, inferomedial screws, and allograft [10]. Many biomechanical studies have reported that the combined use of a locking compression plate (LCP) and fibular allograft could increase the initial stiffness and sustain a higher ultimate failure load [11, 12]; however, only a few comparative clinical studies have been performed to date.

Thus, this study aimed to compare the radiological and clinical outcome between LCP (LCP group) and LCP with fibular allograft (FA group) in the treatment of comminuted PHFs. We hypothesized that elderly patients treated with the LCP and fibular strut allograft would have better outcomes and lower complication rate than those treated with the LCP alone.

## Methods

This study was approved by institutional review boards, and written consent was obtained from all patients. This retrospective study included 60 geriatric patients who underwent an open reduction and internal fixation for comminuted PHFs between January 2014 and May 2017. The inclusion criteria were aged 60 years or older, a minimum of 2 years of follow-up, and a diagnosis of displaced three- and four-part PHFs. Patients with pathological fractures, open fractures, fractures that involved articular split of the humeral head, and associated nerve injuries were excluded.

**Fig. 1:**
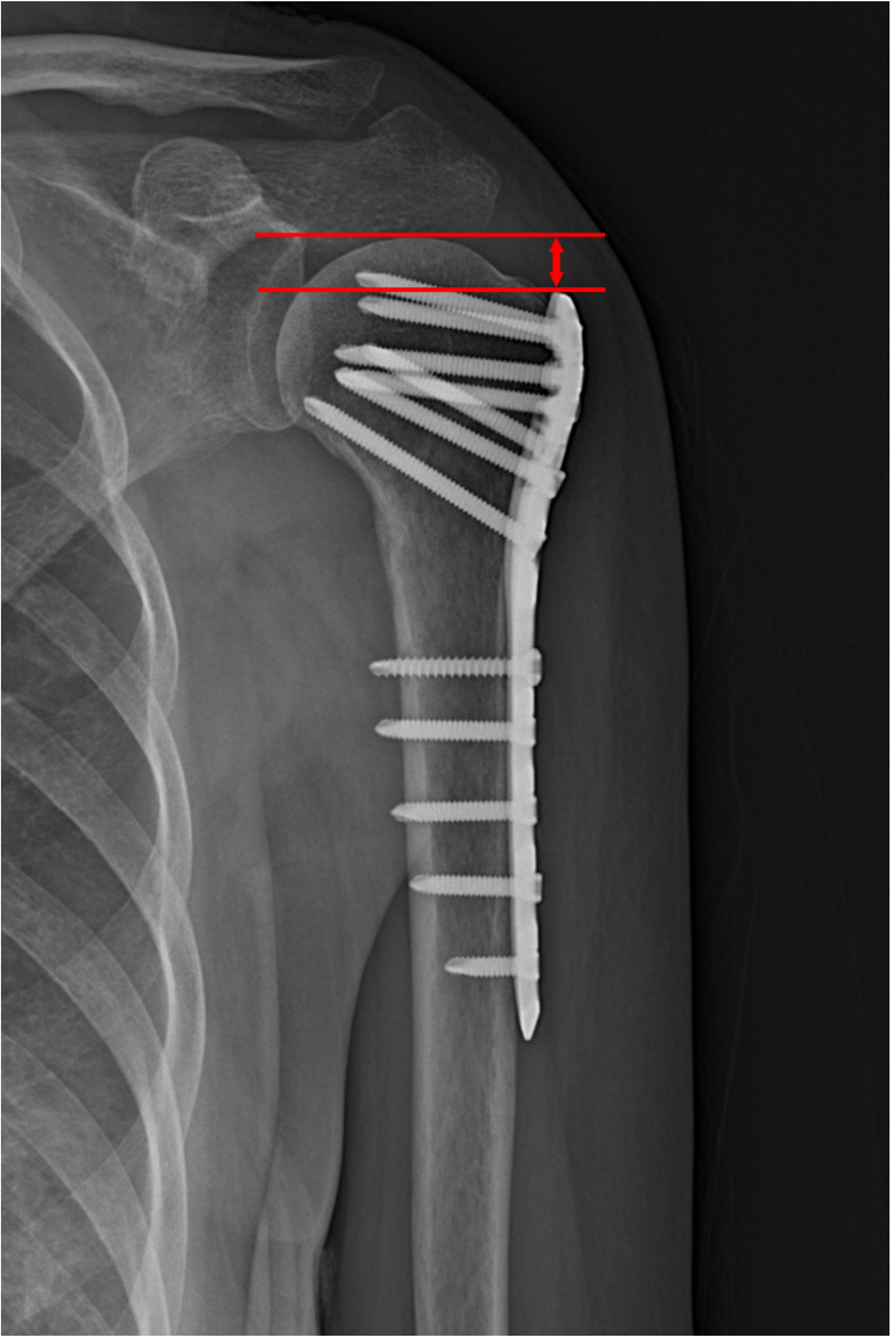
Calculation of the humeral head height. The two lines drawn running perpendicular to the shaft of the plate; one was placed at the top edge of the plate, and the other was placed at the superior edge of the humeral head. The distance between these two lines was measured and designated as the head height

**Fig. 2:**
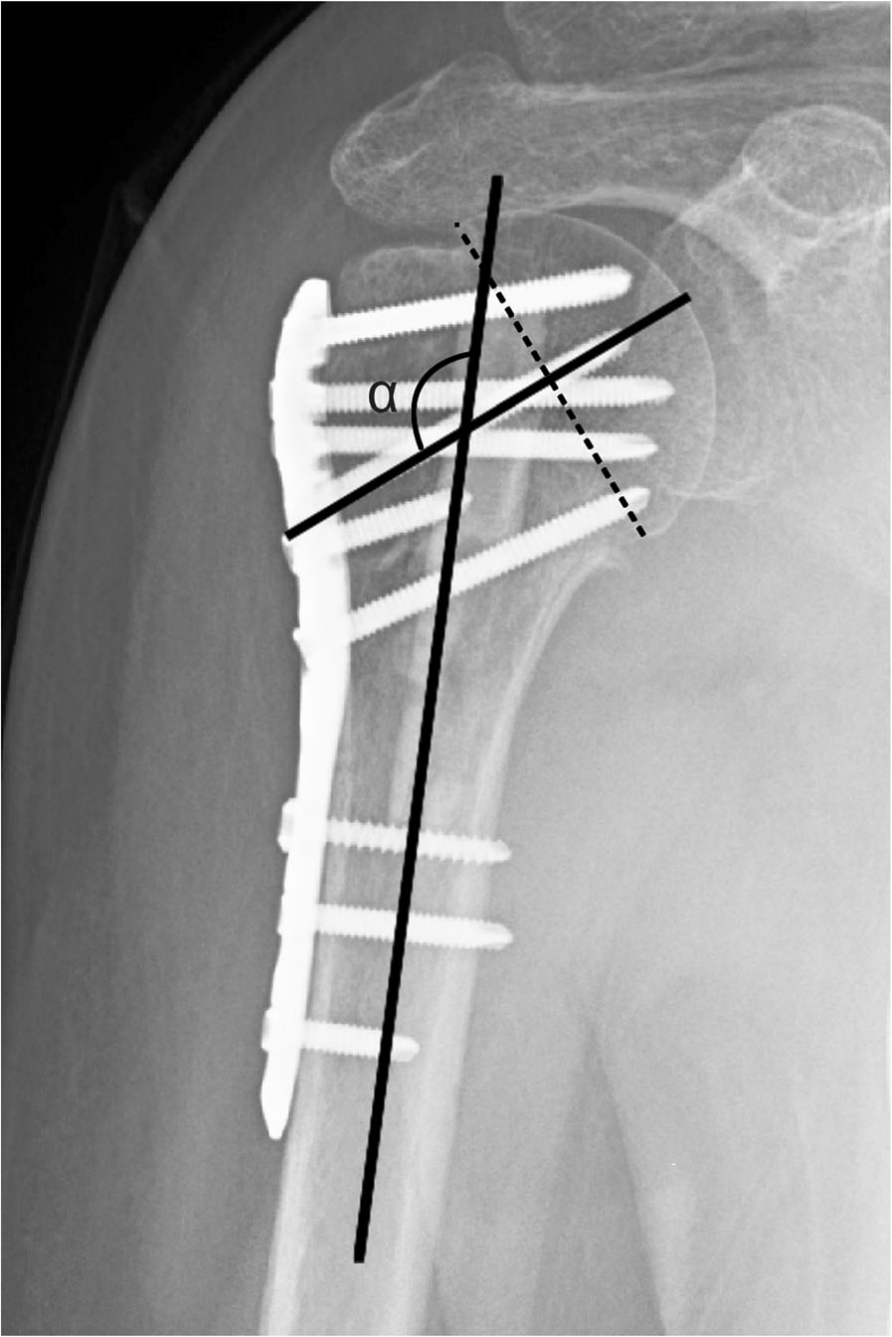
The head-shaft alignment (angle α) was determined as follows: a first line (dashed line) was drawn from the superior border to the inferior border of the articular surface and a second line was drawn perpendicular to the first line through the center of the humeral head. A third line bisected the humeral shaft, and the angle between the second and third line was defined as the head-shaft angle α

Postoperative radiographs were obtained on the second day, 1 month, 3 months, 6 months, 1 year, and 2 years after the surgery. Radiological evaluation was performed by measuring the humeral head height [13] (HHH) and neck shaft angle (NSA) on true anteroposterior (AP) radiographs (Fig. 1). The NSA was measured on AP radiographs with the shoulder in neutral rotation [14]. A line was drawn from the superior to the inferior border of the articular surface. Then, a perpendicular line was drawn through the center of the humeral head. The angle between the perpendicular line and the line bisecting the humeral shaft was defined as the NSA (Fig. 2). A difference in the HHH > 3 mm or the NSA > 5° on the AP radiograph that was taken 2 days after the operation and that obtained at the 2 years follow-up was defined as loss of fixation [15].

Clinical outcomes were evaluated by the American Shoulder and Elbow Society (ASES) [16] score and the Constant-Murley score (CMS) [17]. Shoulder range of motion including forward elevation, external rotation, abduction, and internal rotation was digitally recorded at the first year of follow-up and subsequent yearly evaluation. Complications such as infection, screw perforation, and humeral head necrosis were recorded during follow-up.

### Surgical technique

All patients were positioned in the beach chair position with the injured arms hanging over the edge of the radiolucent operating table. The deltopectoral approach was performed to gain access to the proximal humerus. The humeral head and the fragments were retracted and reduced by placing no. 2 nonabsorbable sutures (Ethicon, Somerville, USA) through the rotator cuff. In the LCP group, anatomical reduction of the fragments was maintained by temporary Kirschner wires and checked by fluoroscopy. Thereafter, a proximal humeral LCP (Synthes, Oberdorf, Switzerland) was placed on the lateral cortex and fixed with cortical and locking screws. The plate was placed 5 mm inferior to the upper end of the greater tuberosity and 1 cm posterior to the bicipital groove [18].

**Fig. 3:**
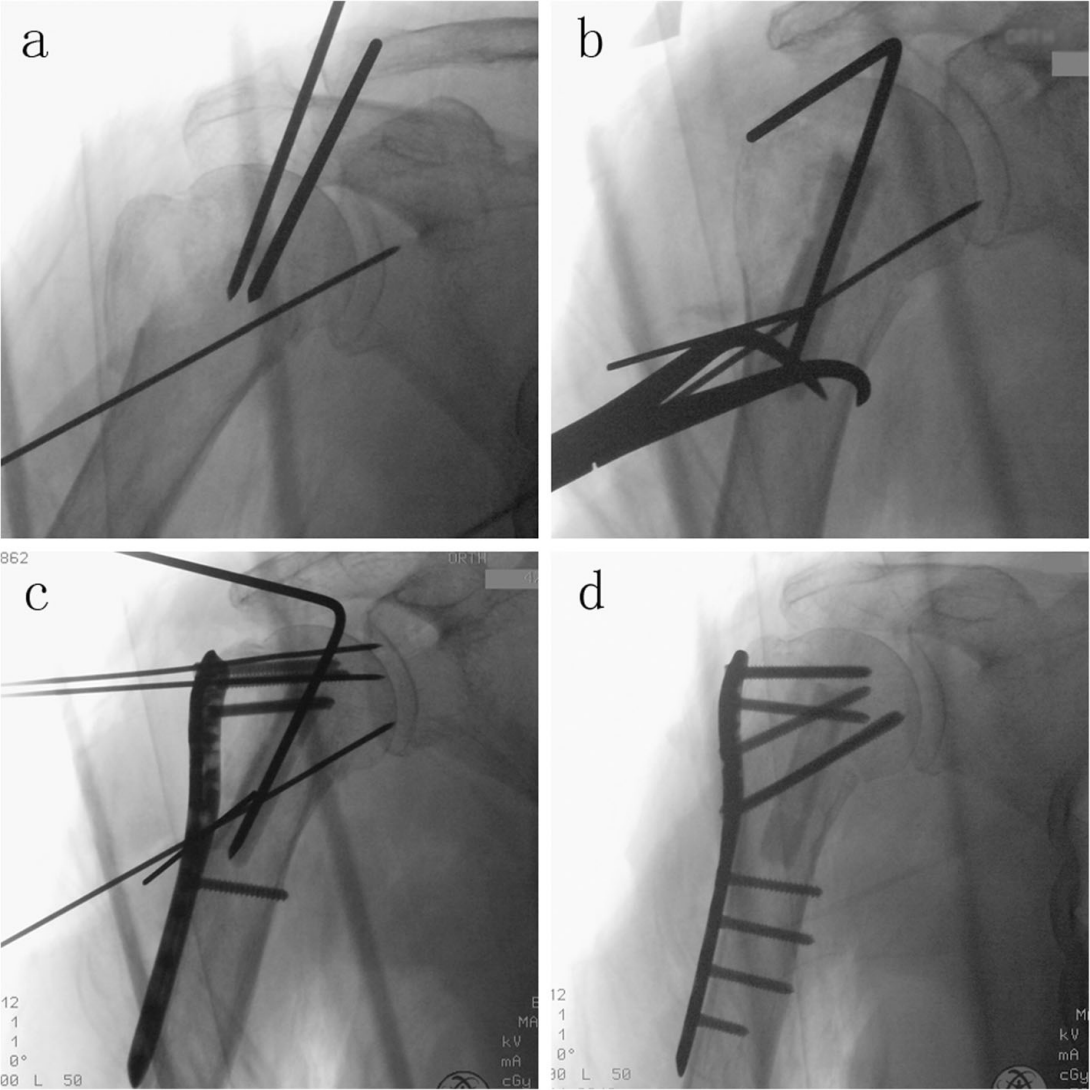
The reduction and fixation process with a fibular strut allograft. **a** A 1.5 mm guidewire was placed 1 cm posterior to the intertubercular groove and 1 cm medial to the transition between the head and the greater tuberosity. **b** The fibular allograft was inserted into the cavity, through the fracture site, through the guidewire. And then, it was pushed upwards to support the humeral head in a proper height. **c** After confirming the fracture reduction, a LCP was used to fix the fragments. Locking screws were placed through the graft into the humeral head and shaft. **d** Post-operative radiograph showing good anatomical reduction

**Fig. 4:**
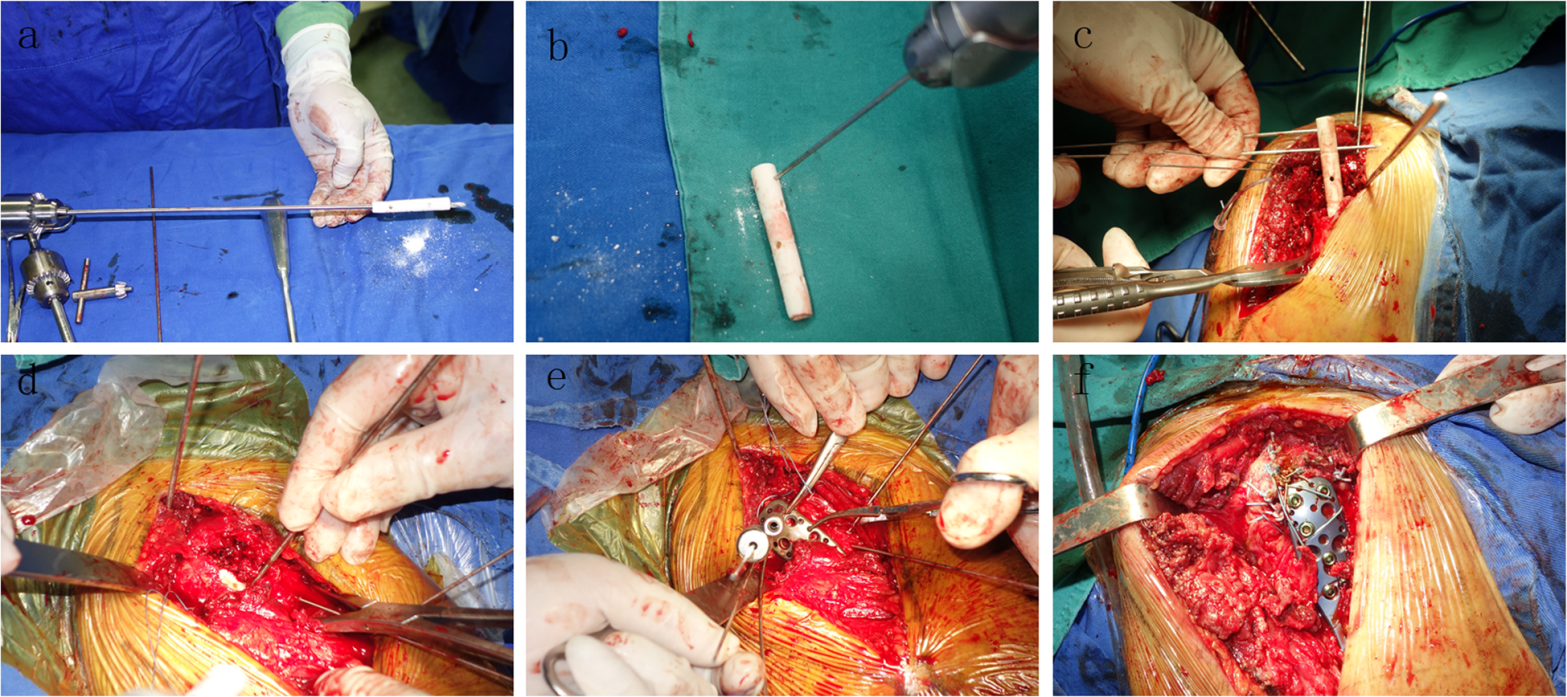
surgical procedure (**a**, **b**) Holes were drilled on the fibular allograft to make the fibula easily maneuvered into position. **c** The fibular allograft was inserted into the cavity through the bone defect. **d** Without the exposure of medial calcar, the medial column could be indirectly reduced to pushing the fibular allograft upwards. **e** A proximal locking plate was used to fix the greater tuberosity to the humeral head. **f** Multiple non-absorbable sutures were passed to compress comminuted fragments to the bony defect of the proximal humerus

In the FA group, the fibular allograft was used to indirectly reduce the fracture. A 1.5-mm guidewire was placed 1 cm posterior to the intertubercular groove and 1 cm medial to the transition between the head and the greater tuberosity. A fibular strut was inserted into the cavity, through the fracture site, and through the guidewire. By this procedure, the proximal fibular allograft was located at the center of the head, and the distal end was positioned in the humeral shaft. The proper height and position of he humeral head could be achieved by pushing the allograft upward instead of medial (Fig. 3). Further, greater tuberosity fragments were reduced and temporarily fixed. A LCP was used to complete the fixation after the fracture reduction was confirmed under C-arm. Locking screws were used to pass through the allograft to lock its position (Fig. 4). Two infer-medial calcar screws were also inserted to provide additional support.

A sling was provided to immobilize the arm during the first 4 weeks after surgery in both groups. Continuous pendulum and passive range-of-motion exercises were started 2 days postoperatively. Active assisted range-of-motion exercises were followed at 4 weeks. The patients could start to do daily activities according to the tolerance of the patients and the fracture union status.

### Statistical analysis

Statistical analysis was performed using SPSS software version 18.0 (SPSS Inc., Chicago, IL, USA). Continuous data and scores for the LCP and FA groups were evaluated with an independent t test. Differences in proportions were compared with a chi-square test and Fisher’s exact test. The threshold for significance was set at *p* < 0.05.

## Results

A total of 60 patients (26 men and 34 women) were included in this study. The LCP group comprised 35 patients, of which 25 patients had three-part fractures and 10 had four-part fractures. The FA group consisted of 25 patients, of which 17 patients had three-part fractures and 8 had four-part fractures. In the LCP group, 18 patients experienced medial comminution, and the mean follow-up period was 32.23 (range, 25–40) months. In the FA group, 13 patients experienced medial comminution, and the mean follow-up period was 31.56 (range, 24–40) months (Table 1).

**Table 1:** Demographic characteristics data for patients included in this study

Characteristic	LCP Group (*n* = 35)	FA Group (*n* = 25)	*P*-value
Average age (year)	72.46	73.16	0.566
Sex distribution (male: female)	11:24	7:18	0.775
Dominant arm involvement	17:18	13:12	0.793
The mean time from injury to surgery (day)	4.69	4.48	0.237
Te mechanism of injury (F: TA)	27:8	20:5	0.791
Classifcation of Neer (3 part: 4 part)	25:10	17:8	0.775
Medial comminution	18:17	13:12	0.965
The mean follow-up period (months)	32.23	31.56	0.898

*LCP* locking compression plate, *FA* fibular allograft

**Fig. 5:**
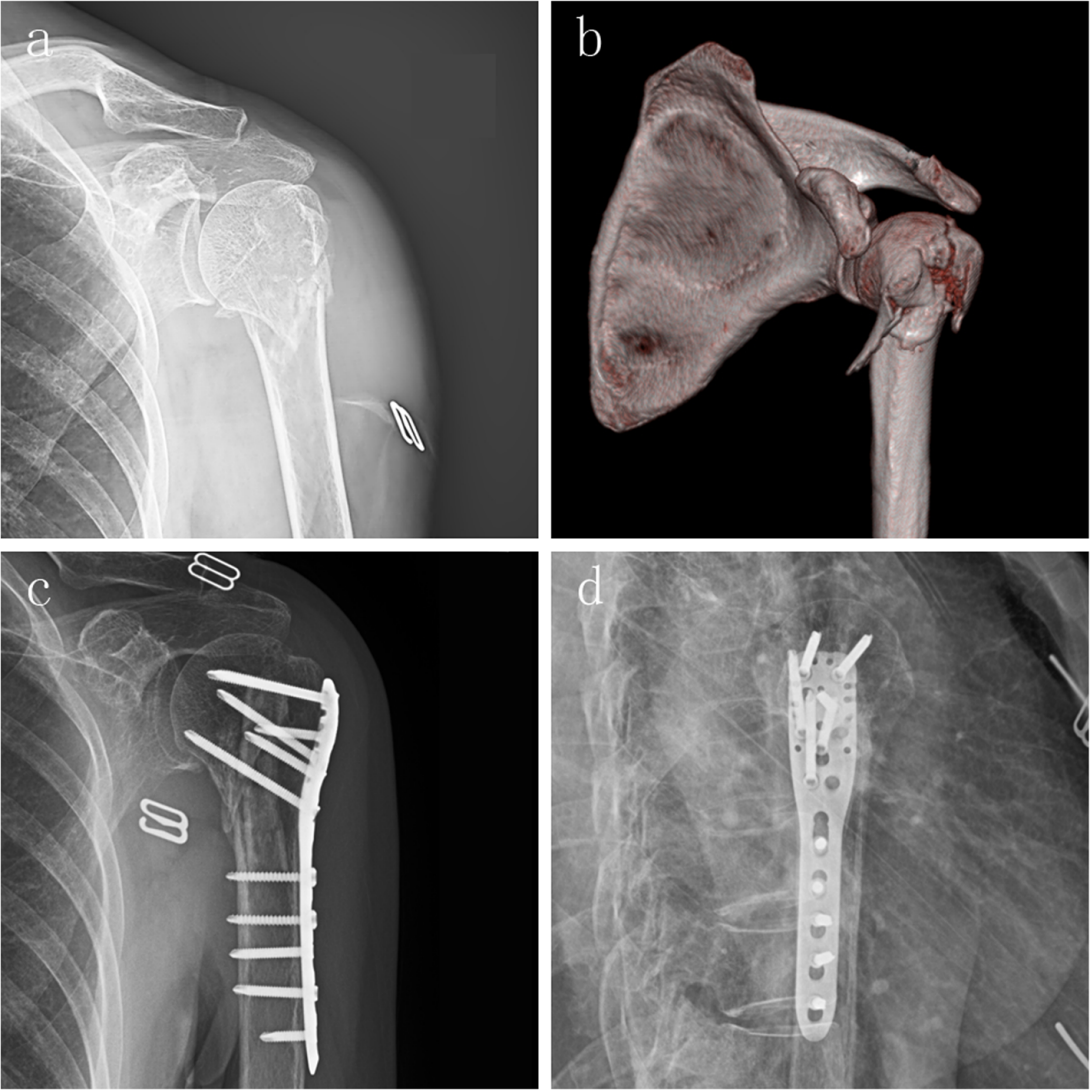
A case of a 3-part fracture with severe metaphyseal comminution. **a** Radiograph of a displaced 3-part humeral fracture in the left shoulder of a 73-year-old woman. **b** CT scan, 3-D reconstruction view. (**c**, **d**) Radiograph 1 year after surgery

In the LCP group, the average change in HHH was 4.16 mm (range, 0–13 mm), a change in HHH > 3 mm appeared in 18 patients. The average change in NSA was 9.94° (range, 0–30°), a change in NSA > 5° appeared in 20 patients. In the FA group, the average change in HHH was 1.18 mm (range, 0–13 mm), a change in HHH > 3 mm appeared in 3 patients. The average change in NSA was 3.12° (range, 0–8°), a change in NSA > 5° appeared in 4 patients (Fig. 5).

**Fig. 6:**
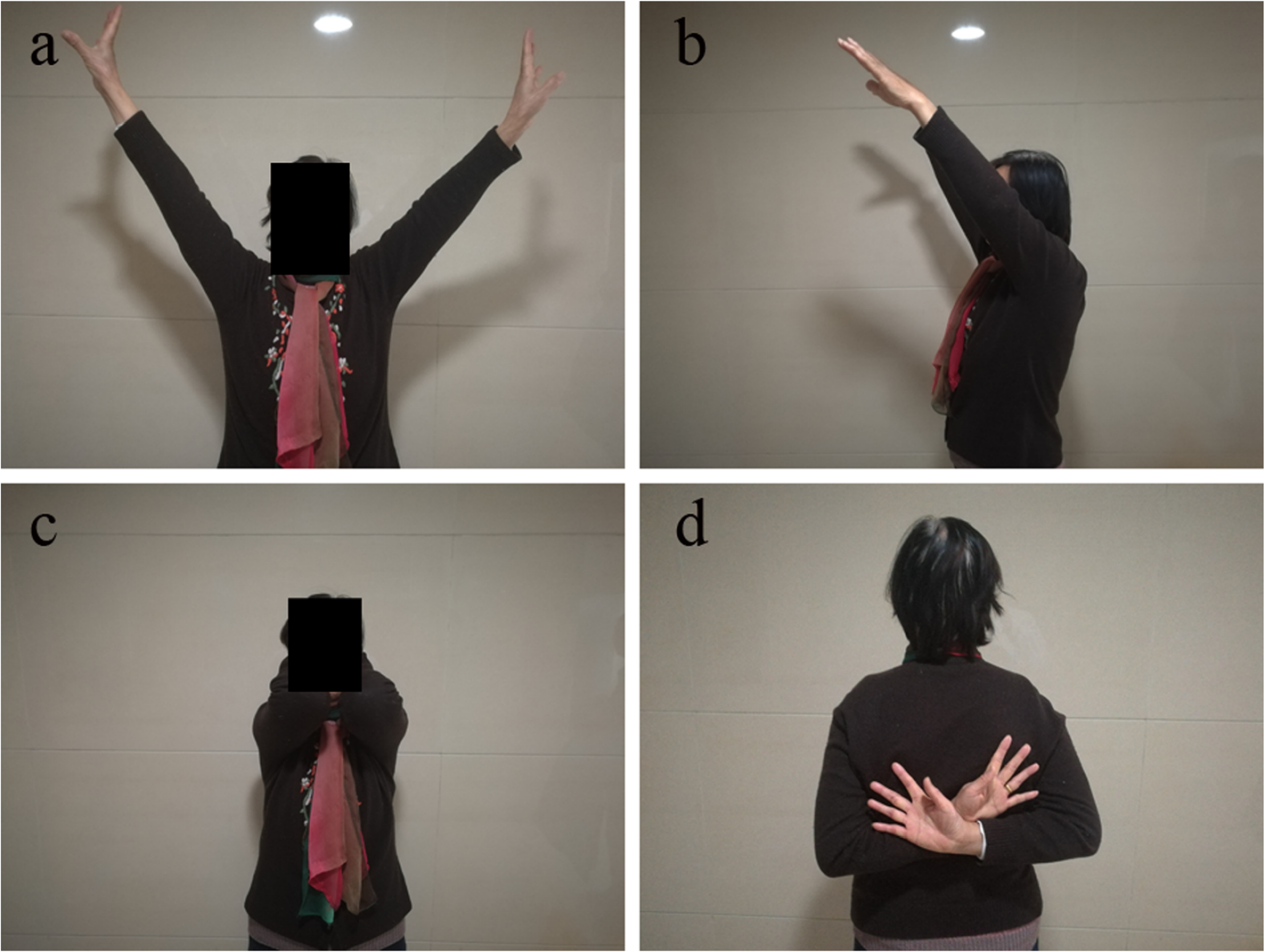
Clinical function 2 years after surgery. **a** Active abduction. **b** Active forward elevation. **c** Active external rotation. *d*. Active internal rotation

The CMS, ASES scores, and shoulder range of motion for all patients are presented in Table 2. Compared with the LCP group, the FA group had significantly better mean CMS (78.96 versus 73.00; *p* = 0.024) and ASES scores (78.64 versus 72.80; *p* = 0.022). At 2 years, the active forward elevation, abduction, external rotation, and internal rotation of the shoulder were 128.49 ± 22.81°, 122.37 ± 22.31°, 55.09 ± 8.63°, and L1, respectively, in the LCP group compared with 144.04 ± 21.37°, 140.64 ± 20.34°, 58.96 ± 8.49°, and T12 in the FA group (Fig. 6). Significant difference in active forward elevation (*p* = 0.010) and abduction (*p* = 0.002) of the shoulder was found between the two groups. No significant difference in the mean external rotation values (*p* = 0.090) and internal rotation values (*p* = 0.438) was noted between the two groups. There were 12 complications in 10 of 35 patients (28.57%) in the LCP group, including varus malunion in 5, screw penetration in 5, and avascular necrosis in 2. In the FA group, one patient presented screw penetration, and two patients developed avascular necrosis of the humeral head.

**Table 2:** Radiographic Evaluation, Outcome Scores and Range-of-Motion Data for the Study Population

Variable	LCP Group (*n* = 35)	FA Group (*n* = 25)	*P*-value
HHH (mm)^a^	4.16 ± 4.2	1.18 ± 1.08	< 0.001
NSA^a^	9.94 ± 9.92°	3.12 ± 3.13°	< 0.001
CMS^a^	73.00 ± 9.94	78.96 ± 9.71	0.024
ASES^a^	72.80 ± 9.73	78.64 ± 9.18	0.022
Forward elevation^a^	128.49 ± 22.81°	144.04 ± 21.37°	0.010
Abduction^a^	122.37 ± 22.31°	140.64 ± 20.34°	0.002
Internal rotation^b^	L1(buttock-T5)	T12 level (L5-T5)	0.438
External rotation^a^	55.09 ± 8.63°	58.96 ± 8.49°	0.090

*LCP* locking compression plate, *FA* fibular allograft, *HHH* humeral head height, *NSA* neck shaft angle, *ASES* American Shoulder and Elbow Society score, *CMS* Constant-Murley score

^a^The values are given as the mean and standard deviation. ^b^The values are given as the mean with the range in parentheses. °degree

## Discussion

This study compared the LCP group and the FA group involving three- and four-part PHFs in patients aged 60 years or older and evaluated the influence of fibular allograft on the radiological and clinical outcomes. PHFs treated by LCP and fibular allograft showed significantly better clinical outcomes, and a lower complication rate. The FA group also showed superior radiological results regardless of the fracture type.

The treatment of unstable and displaced PHFs in elderly patients remains a challenge [19]. Anatomical reduction is difficult to maintain because of the poor quality of the humeral head, and many surgeons believe that LCP fixation is a promising treatment for this problem [20]. Compared to the standard nonlocking plate, the screw fixation angle can be oriented in different directions when an LCP is used, and the locking screw provides stable fixation maintenance [21]. However, many studies have reported variable outcomes, with high rates of complications, including screw penetration, varus collapse, and avascular necrosis of the humeral head, especially in older patients with osteoporosis or medial column comminution [22, 23]. Ockert et al. [24] reported 10-year outcomes after operative treatment with LCP for unstable and displaced PHFs, and the majority of the patients obtained good or excellent outcomes. However, poor outcomes and complications were found in older and female patients. In recent years, many efforts have made to overcome these problems. Clinical and biomechanical studies have paid increased attention to allograft augmentation to increase the stability of locking plate fixation in PHFs. Gardner et al. [25] were the first to introduce this technique using fibular strut allograft to indirectly reduce the fracture and maintain the fixation; in their study, all of the seven fractures achieved complete union without loss of fixation.

Mathison et al. [12] first made a biomechanical comparison between locking plate alone and locking plate with fibular allograft. They created a 10-mm wedge-shaped osteotomy at the lever of the surgical neck to simulate the comminution of the medial column. Load-displacement curve was used to test failure load and stiffness of the constructs. Their study demonstrated that the bone peg increased the failure load and the initial stiffness of the constructs. Relative to locking plate fixation alone, failure load was increased by 1.72 times and the initial stiffness was increased by 3.84 times. Chow et al. [11] performed a similar study to evaluate the effect of a fibular allograft. No augmented construct collapsed before 25,000 cycles, while most of the specimens in the non-augmented locking compression group collapsed at mean 6604 cycles. Neviaser et al. [26] retrospectively reviewed 38 patients treated by locking plate with endosteal strut augment, and they reported high clinical outcome scores and lower complication rate. Recently, Panchal et al. [27] assessed the effect of intramedullary fibular allograft on the clinical and radiological outcomes in unstable PHFs with medial column disruption. According to the clinical rating scale, 26 patients had excellent or good outcomes, six patients showed fair outcomes, and only four patients experienced poor outcomes. With regard to the restoration of the humeral NSA, the result was good in 31, fair in three, and poor in two cases. When calculating the HHH, the average loss of reduction was measured as 1.6 mm. Only two cases experienced varus collapse of the humeral head, and osteonecrosis was noted in two patients. Cha et al. [15] compared the radiological outcomes of using only LCP and using LCP with fibular allograft in the treatment of comminuted PHFs. The change in the NSA and HHH in the LCP group was markedly higher.

In our opinion, the fibular allograft was a reasonable option to maintain the anatomical reduction in the treatment of comminuted PHFs in the elderly patients. The fibular allograft could be used as tool to indirectly reduce the fracture. Gardner et al. [25] first introduced the use of screw to push the fibular allograft medially so that the graft could indirectly reduce the fractured medial cortex. Subsequently, many authors preferred to push the graft upward instead of medial because it could support the humeral head in a proper height [15, 28]. However, in some cases, the intramedullary cavity in elderly patients accompanied osteoporosis was relatively large, the graft could not be easily manipulated. So we placed a guide pin at the apex of the humeral head. Then, the fibular allograft was pushed upward in the intramedullary cavity through the guide pin as in retrograded nailing. Especially in cases with medial cortex disruption, using fibular allograft as a pillar to support the humeral head from intramedullary cavity was more helpful in maintaining reduction. The added stability provided by the fibular allograft allowed for an early rehabilitation program and reduced the complication rate. In our study, the FA group showed significant lower rates of varus malunion and screw penetration. The fibular allograft also had disadvantages, such as risk of infection, disease transmission, and high cost. The fibular allograft contains cortical bone, so it might be fractured during insertion of the screws.

This study has several limitations. First, it was a retrospective study, and the number of patients was relatively small. Further study with a greater number of patients is needed. Second, the follow-up duration was rather short, as the difference in NSA and HHH might change with longer monitoring. Third, the Neer classification is the most widely used grading system for PHFs, but some studies have shown that the Neer classification only have fair to good reliability.

## Conclusion

Elderly patients treated with an LCP and fibular allograft achieved better radiological outcomes, clinical outcomes, and a lower rate of complications compared with those who treatment with an LCP alone for the treatment of a three- or four-part PHFs. Using the fibular allograft is a reasonable option to help the reduction, provide additional support to the humeral head, improve outcomes, and minimize complications.

## Data Availability

The datasets used and analysed during the current study available from the corresponding author on reasonable request.

## Abbreviations

AP: Anteroposterior
ASES: American shoulder and elbow society
CMS: Constant-murley score
FA group: Locking plate with fibular allograft
HHH: Humeral head height
LCP: Locking compression plate
NHA: Neck-shaft angle
PHF: Proximal humeral fractures
